# Le biote du Canada: les arthropodes terrestres

**DOI:** 10.3897/zookeys.819.31830

**Published:** 2019-01-24

**Authors:** David W. Langor, Cory S. Sheffield

**Affiliations:** 1 Ressources naturelles Canada, Service canadien des forêts, 5320 – 122 St. NW, Edmonton, Alberta, T6H 3S5, Canada Natural Resources Canada, Canadian Forest Service Edmonton Canada; 2 Royal Saskatchewan Museum, 2340 Albert Street, Regina, Saskatchewan, S4P 2V7, Canada Royal Saskatchewan Museum Regina Canada

*Cet ouvrage est dédié aux visionnaires qui ont conçu et créé la Commission biologique du Canada.*

Il y a quarante ans, un groupe de scientifiques visionnaires, principalement composé d'entomologistes et d'arachnologues, a entrepris un ambitieux projet visant à produire la monographie *Canada and its insect fauna* (Danks 1979). Cette publication historique a été le remier grand ouvrage scientifique réalisé par la Commission biologique du Canada (CBC) peu de temps après sa création. Hugh V. Danks était responsable de la coordination de la vaste équipe et de l'ambitieux plan de travail ainsi que de l'édition de la publication. La réputation de Hugh pour sa rigueur scientifique, son sens de l'organisation, son souci du détail et son esprit de synthèse est légendaire et inspirante. Ses qualités se sont révélées essentielles au succès du projet. En 573 pages imprimées, cette équipe de 60 spécialistes a examiné les tendances et les déterminants de la diversité régionale, les habitats des insectes aquatiques et terrestres, les méthodes de documentation de la faune ainsi que le statut de chaque groupe d'arthropodes terrestres au Canada. Le reste appartient à l'histoire! La publication constitue la référence pour la science de la biodiversité au Canada depuis quatre décennies. L'ouvrage intégral et ses chapitres individuels ont été cités des milliers de fois, ce qui témoigne l’influence notable de cette publication au Canada et ailleurs. En outre, *Canada and its insect fauna*, tout comme les nombreuses autres publications de la CBC qui ont suivi sur près de quatre décennies, prouve que de grandes réalisations peuvent être accomplies par des communautés d'individus dévoués, compétents et passionnés ayant la volonté de surmonter des obstacles institutionnels parfois contraignants afin de réaliser quelque chose de remarquable et de durable. Cette approche scientifique exceptionnelle continue à définir la CBC.

Même après près de 40 ans, le contenu de *Canada and its insect fauna* est extrêmement précieux. Néanmoins, une partie du contenu des 39 chapitres relatifs à la faune, notamment les informations contenues dans les tableaux, devait être mise à jour afin de tenir compte des progrès réalisés depuis 1979. Ainsi, la CBC a commencé à planifier un projet de mise à jour de ces chapitres en 2016. On s'est cependant vite rendu compte que la nécessité de réévaluer l'état des connaissances sur la diversité des espèces au Canada dépassait le cadre des arthropodes terrestres pour couvrir l'ensemble du biote, la dernière évaluation ayant été publiée en 1995 (Mosquin et al. 1995). Un certain nombre d’autres organismes ont été consultés et ont approuvé le concept d'une nouvelle évaluation de la biodiversité pour le Canada. Le projet Biota of Canada a donc été lancé dans le but de passer en revue et d'évaluer l'état des connaissances sur la biodiversité de tous les groupes d'organismes au Canada en publiant une série de volumes. Les arthropodes terrestres constituaient un point de départ évident pour élaborer la «démonstration de faisabilité», puisque le 40^e^ anniversaire de la CBC approchait à grands pas et ce travail d'envergure devait servir à commémorer l'occasion.

Un comité de rédaction a été mis sur pied au sein de Biota of Canada afin d'élaborer des lignes directrices relatives à la préparation des manuscrits et de garantir une approche normalisée des sujets traités et des données fournies. Soutenu par un ensemble de «directives aux auteurs» et un exemple de manuscrit à distribuer aux auteurs potentiels, le processus de recherche des auteurs principaux s'est déroulé de manière remarquablement fluide. Presque tous les auteurs initialement sollicités ont accepté de se joindre à l'équipe de rédaction, ce qui montre à quel point le projet Biota of Canada était perçu comme ayant une grande valeur. La rédaction de la plupart des manuscrits a été dirigée par des spécialistes travaillant dans des établissements canadiens, mais il n'existait aucun expert canadien actuel ou disponible pour dix groupes d'organismes; des experts ont donc été recherchés et retenus dans d'autres pays (Chine, Norvège, États-Unis, République tchèque). Ainsi, Biota of Canada est un effort international.

Tous les auteurs ont été invités à examiner l'état des connaissances sur la diversité de leur taxon au Canada, en portant une attention particulière aux progrès réalisés depuis la publication de *Canada and its insect fauna* (1979). Chaque auteur a été chargé de produire un tableau fournissant pour chaque famille les informations suivantes sur la faune canadienne: le nombre d'espèces signalées en 1979, le nombre d'espèces décrites actuellement connues, le nombre de DNA Barcode Index Numbers (BINs; Ratnasingham et Hebert 2013) attribués aux spécimens canadiens figurant dans la base de données Barcode of Life Datasystems (BOLD; Ratnasingham et Hebert 2007), le nombre d'espèces supplémentaires qui devraient éventuellement être découvertes au Canada, leur répartition générale par écozone (Gouvernements fédéral, provinciaux et territoriaux du Canada 2010), et toute importante source d'information ayant fait progresser notre compréhension de la faune depuis 1979. Les auteurs ont également été invités à souligner les lacunes et les occasions importantes en matière de connaissance de la faune canadienne. Au-delà de quelques exigences normalisées, les auteurs étaient libres de rédiger leurs manuscrits comme il leur semblait approprié. Certains ont fourni plus de détails que d'autres ou des analyses zoologiques supplémentaires. Ensemble, ces documents offrent un aperçu complet de l'état des connaissances sur la biodiversité des arthropodes terrestres au Canada. L'ensemble des travaux est analysé et résumé dans un document de synthèse inclus.

Certains groupes inclus dans *Canada and its insect fauna* ont été exclus des travaux en cours, à savoir les tardigrades (oursons d'eau), qui ne sont pas des arthropodes, et les crustacés. Les crustacés terrestres et d'eau douce ont été traités dans deux chapitres de Danks (1979) intitulés «Pentastomida» et «Crustacea»; cependant, comme la grande majorité des crustacés sont marins, ce sous-phylum sera traité dans un futur volume incluant tous les autres animaux du Canada.

Cet ouvrage devrait servir à plusieurs fins. Il y a lieu de se réjouir que nos connaissances sur la faune d'arthropodes terrestres au Canada aient considérablement progressé au cours des 40 dernières années, quoique de façon inégale pour certains groupes. Il est clair que plusieurs décennies de travail ciblé seront nécessaires avant que notre faune soit bien documentée, c'est-à-dire comparable à l'état des connaissances de la plupart des pays européens. Nous nous attendons toutefois à ce que cette évaluation ait une valeur immédiate pour appuyer notre exigence nationale de production de rapports sur de la biodiversité au Canada. Nous espérons que les analyses des lacunes et des besoins contribueront à guider les décideurs dont le mandat est de documenter et de préserver la biodiversité canadienne et d'en faire rapport afin que les ressources futures soient investies de manière appropriée pour en maximiser les avantages. Enfin, ce travail représente un merveilleux exemple de l'esprit de collaboration bien vivant parmi les scientifiques œuvrant dans le domaine de la biodiversité au Canada. Nous espérons que cela inspirera d'autres à contribuer à compléter la série de publications ambitieuses de Biota of Canada.

Le comité de rédaction de Biota of Canada (Robb Bennett, Jeremy deWaard, José Fernández-Triana, Rémi Hébert, David Langor, Jade Savage et Cory Sheffield) a guidé l'orientation stratégique de ce projet. Les rédacteurs tiennent à remercier Jeremy deWaard d'avoir révisé ou fourni les données de codes à barres d'ADN pour presque tous les articles ainsi que le personnel de Pensoft, en particulier Yordanka Banalieva, Plamen Pankov et Veselin Kostadinov, pour leur grande aide dans le processus de révision et de publication. Toute l'équipe de rédaction remercie les nombreux réviseurs experts pour leurs précieux commentaires qui ont permis d'améliorer les articles individuels et, de ce fait, le travail dans son ensemble. Le financement de la publication de ce travail a été fourni par les établissements respectifs des auteurs ainsi que par la CBC.
